# A second hit somatic (p.R905W) and a novel germline intron-mutation of *TSC2* gene is found in intestinal lymphangioleiomyomatosis: a case report with literature review

**DOI:** 10.1186/s13000-021-01138-8

**Published:** 2021-08-31

**Authors:** Bogyeong Han, Juhwan Lee, Yoon Jin Kwak, Hyun-Young Kim, Kwang Hoon Lee, Yumi Shim, Hyunju Lee, Sung-Hye Park

**Affiliations:** 1 Department of Pathology, Seoul National University Hospital, Seoul National University College of Medicine, 103 Daehak-ro, Jongno-gu, Seoul, 03080 South Korea; 2 School of Electrical Engineering and Computer Science, Gwangju Institute of Science and Technology, 123 Cheomdangwagi-ro, Buk-gu, Gwangju, 61005 Republic of Korea; 3 Department of Surgery, Seoul National University Hospital (SNUH), Seoul National University College of Medicine, Seoul, Republic of Korea; 4 Center for Medical Innovation, Seoul National University Hospital, Seoul, Republic of Korea; 5 Department of Pathology, Seoul National University College of Medicine, Seoul, 03080 Republic of Korea

**Keywords:** Intron retention, Lymphangioleiomyomatosis, Germline mutation, Somatic mutation, Perivascular epithelioid cell tumor (PEComa), Tuberous sclerosis complex

## Abstract

**Background:**

Tuberous sclerosis complex (TSC) is an autosomal dominant disorder characterized by hamartomas in multiple organs associated with germline mutations in *TSC1* and *TSC2,* including exonic, intronic, or mosaic mutations. Gastrointestinal (GI) tract Lymphangioleiomyomatosis (LAM) is an extremely rare manifestation of TSC, with few reported cases. Herein, we aimed to determine the driver mutation, pathogenesis, and relationship of germline and somatic mutations of LAM through whole-genome sequencing (WGS) of the tumor and blood samples and whole transcriptome sequencing (WTS) analysis.

**Case presentation:**

A nine-year-old girl with a full-blown TSC presented with abdominal masses detected during a routine check-up. Resected intestinal masses were diagnosed as LAM by thorough pathological examination. Interestingly, the LAM presented a somatic *TSC2* gene mutation in exon 24 (p.R905W, c.C2713T), and the patient had intron retention by a novel germline mutation in the intron region of *TSC2* (chr16:2126489, C > G).

**Conclusion:**

Our case suggests that intron retention by a single nucleotide intronic mutation of *TSC2* is sufficient to develop severe manifestations of TSC, but the development of LAM requires an additional somatic oncogenic mutation of *TSC2*.

**Supplementary Information:**

The online version contains supplementary material available at 10.1186/s13000-021-01138-8.

## Background

Herein, we report a case of Tuberous sclerosis complex (TSC) with hamartomas in multiple organs and gastrointestinal lymphangioleiomyomatosis (GI LAM). TSC is an autosomal dominant disorder characterized by hamartomas in multiple organs, seizure disorders, mental retardation, and a reported prevalence of 1/6000 to 1/10,000 live births [1, 2]. *TSC1* (9q34) and *TSC2* (16p13), both known tumor suppressors, are the causative genes. Mutations in these genes result in dysregulation of the mammalian target of rapamycin (mTOR pathway) [3]. To date, more than 1000 mutations have been reported in *TSC1* and *TSC2* [4]. However, 10–15% of clinically diagnosed TSC have no known identifiable mutations and could be attributed to mutations in the promoter, enhancer, or intron region, as well as mosaicism or technical problems [4–6]. Tyburczy et al. have identified mutations in 85% (45 of 53) of patients with TSC in whom no mutations were initially identified by conventional testing [6]. Most of these mutations can be attributed to mosaicism (58%) and intronic mutations (40%) [6]. TSC patients with mosaicism tend to present less severe symptoms, consistent with the gene dosage effect [5]. Intronic mutations are rare and remain undetected with whole-exome sequencing (WES) and with whole-genome sequencing (WGS), special analysis algorithms are still required.

Lymphangioleiomyomatosis (LAM) is a rare neoplasm. Notably, it is a type of perivascular epithelioid cell tumor (PEComa), characterized by aberrant proliferation of smooth muscle-like cells that express melanocytic (HMB45 and Melan-A) and smooth muscle markers (smooth muscle actin, desmin, and caldesmon), as well as melanocyte inducing transcription factor/ Microphthalmia-Associated Transcription Factor (MITF) [7]. There are two identified subtypes: sporadic-LAM (S-LAM) and TSC-related LAM (TSC-LAM). Both are induced by *TSC1* or, more commonly, *TSC2* gene mutations [8]. The gastrointestinal (GI) tract is a rare site for LAM.

By employing WGS and transcriptome studies, we detected germline intronic and somatic *TSC2* gene mutations in this patient. Typically, intron retention has been considered “noise” as it is technically challenging to detect and quantify transcripts with retained introns globally [9]. However, we hypothesize that intron retention of the TSC2 protein by intron mutation could be a driver mutation of TSC and that *TSC2* somatic mutation is responsible for the development of LAM. The percentage of *TSC1/TSC2* second-hit tumors is 65.1% of TSC-related tumors [10]. Herein, we focus on the tumorigenic mechanism of intron retention and second hit somatic mutation of *TSC2* observed in LAM.

## Case presentation

A nine-year-old girl presented with abdominal masses detected in an abdominal sonogram during follow-up check for her TSC manifestations and any tumor development.

**Fig. 1:**
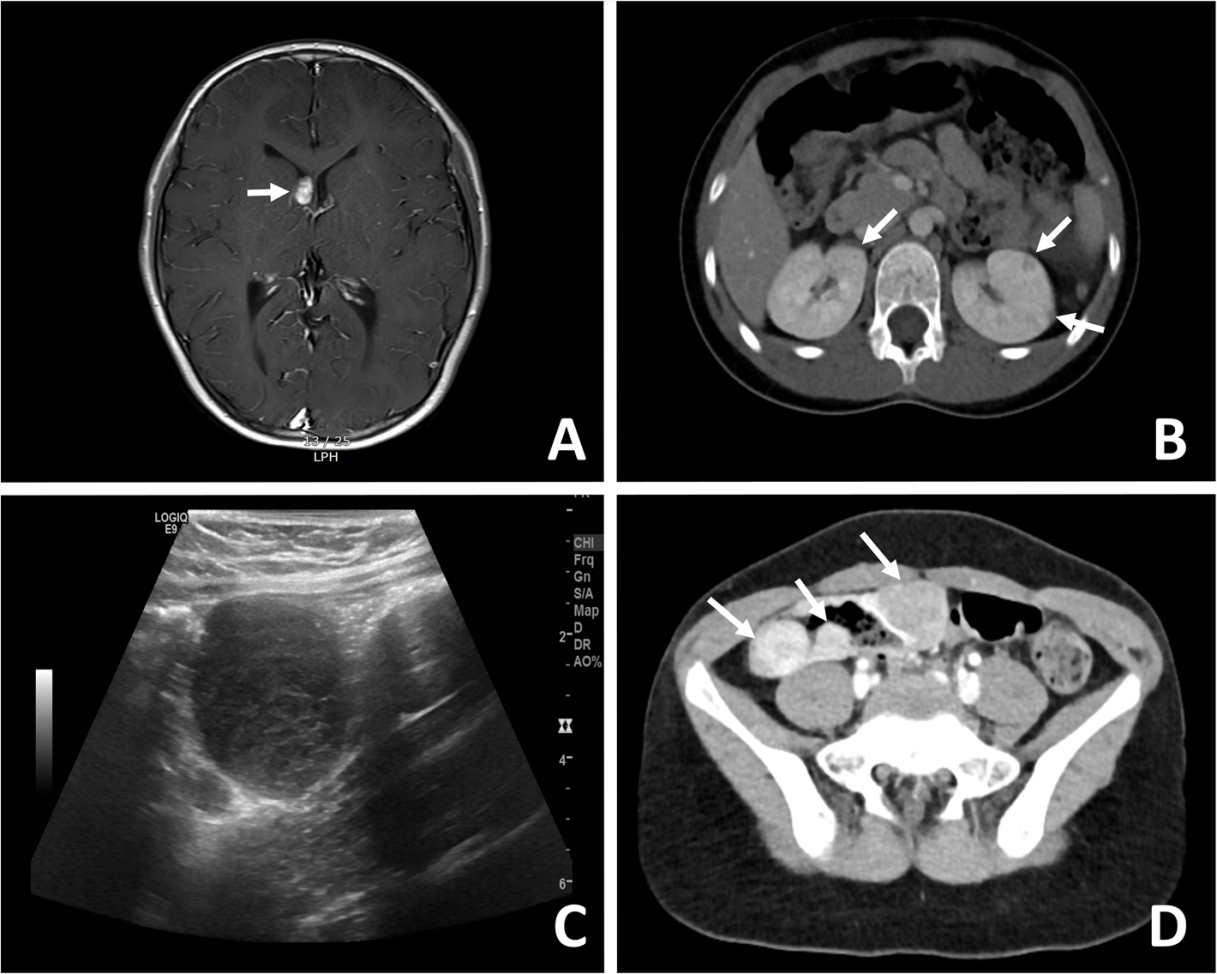
**A** Brain MRI showing an ovoid, enhancing mass at the right lateral ventricle (arrow). **B** Abdominal CT scan showing multiple low-attenuated foci in both kidneys (arrows). **C** Ultrasonography findings showing multiple low echoic nodules along the bowel wall. **D** Abdominal CT scan showing multiple enhancing masses along the ascending colon wall (arrows). MRI, magnetic resonance imaging; CT, computed tomography

Prenatal ultrasound first detected nodules in her heart. Under possibility of TSC, ophthalmology and brain MRI were performed after birth and revealed hamartomas in eyes and intraventricular nodules, suggesting subependymal giant cell astrocytoma (SEGA). She was diagnosed with TSC through genetic test conducted at outside hospital. Myoclonic seizures were observed when she was approximately 100 days old. Furthermore, she presented mental retardation and intermittent febrile or afebrile generalized tonic-clonic seizures, with an abnormal electroencephalogram. She was prescribed an antiepileptic drug and was under close observation. Abdominopelvic and chest computed tomography scans showed multiple small nodules in both kidneys, lungs, and intestines, from the cecum to the ascending colon (Fig. 1). She then underwent laparoscopy-assisted right hemicolectomy. No events related to GI LAM occurred during the 2-year postoperative follow-up period.

### Pathology and immunohistochemistry

**Fig. 2:**
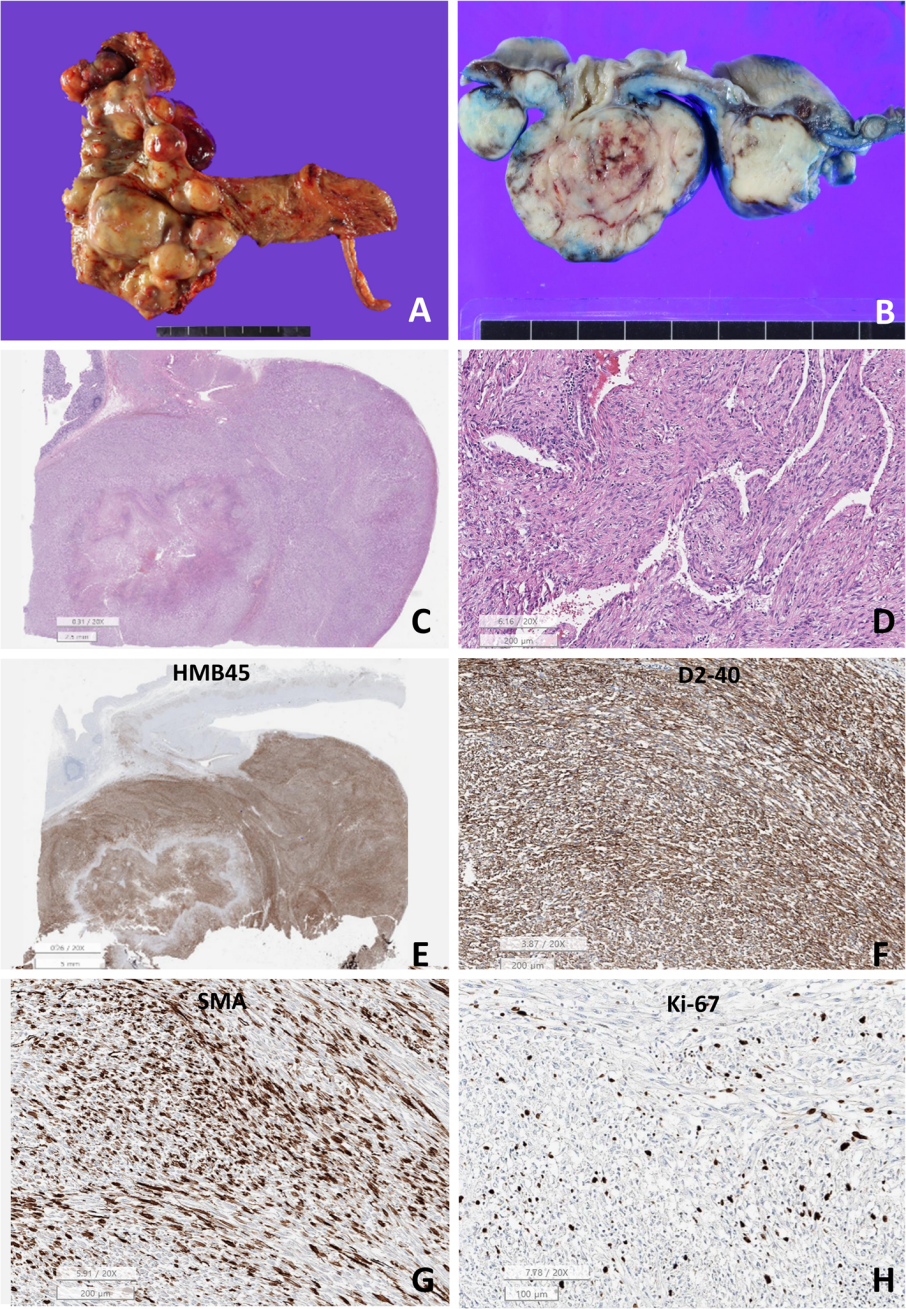
**A** The tumor is well-demarcated, with masses along the intestinal wall. **B**, **C** On sectioning and low power view, the tumor appears to replace the proper muscle layer of the cecum with central suppurative necrosis. **D** High power view of the tumor shows spindle shape mesenchymal cells with slit-like lymphatic channels. The tumor cells show little nuclear atypia. **E**, **F** The tumor cells are positive for HMB45 and D2–40. **G** Tumor is focally positive for smooth muscle actin (SMA). **H** Ki-67 index is 7.0%. Above mentioned pathological and immunohistochemical features are consistent with LAM. (**A**, **B**: gross picture, **C**, **D**: H&E, **E**: HMB45, **F**: D2–40, **G**: SMA, **H**: Ki-67, lower bar: C = 2.5 mm, D = 200 μm, E = 5 mm, F,G: 200 μm, H = 100 μm). LAM, lymphangioleiomyomatosis

On gross examination, multiple nodules were observed along the cecum wall, up to the ascending colon. The largest nodule was 6.1 cm (Fig. 2). The nodules were well-demarcated, tan-to-white, and had solid consistency, with hemorrhage and necrosis. Microscopically, the tumor was composed of smooth muscle-like spindle cells, with bland-looking elongated nuclei and eosinophilic cytoplasm arranged in a fascicular pattern. Tumor cells were distributed around the slit-like vascular spaces lined by flattened endothelial cells. Focal necrosis was observed in the center of the largest nodule, and the mitotic index was 2/50 high power fields. In total, 4 out of the 17 lymph nodes presented metastatic LAM.

Immunohistochemical staining was performed on an immunostaining system (BenchMark ULTRA system, Ventana-Roche, Mannheim, Germany) using primary antibodies, including HMB45 (A: 200, Millipore, Temecula, US), D2–40 (1: 50, DAKO, Glostrup, Denmark), smooth muscle actin (SMA, 1:500, DAKO), c-kit (CD117, 1: 500, Abcam, Cambridge, UK), CD34 (1:200, DAKO), caldesmon (1: 1000, DAKO), desmin (1:200, DAKO), myogenin (1: 500, DAKO), Melan-A (1: 1000, Cell Marque, Rocklin, USA), and Ki-67 (1: 100, DAKO) (Table 1). Appropriate positive controls were included, and primary antibodies were omitted from negative controls.

**Table 1:** Primary antibodies used in this study

Antibody	Dilution	Antigen retrival	Source
HMB45	1: 200	Ventana CC1 100 °C	Milipore, Temecula, US
Melan-A (Mart-1)	1: 1000	Ventana CC1 100 °C	Cell Marque, Rocklin, US
D2–40	1: 50	Ventana CC1 100 °C	DAKO, Glostrup, Denmark
Phosphorylated mTOR	1: 100	Ventana CC1 100 °C	Cell signaling, Boston, US
C-Kit	1: 500	Ventana CC1 100 °C	Abcam, Cambridge, UK
SMA	1: 500	Ventana CC1 100 °C	DAKO, Glostrup, Denmark
CD34	1: 200	Ventaan CC1 100 °C	DAKO, Glostrup, Denmark
Caldesmon	1: 1000	Ventaan CC1 100 °C	DAKO, Glostrup, Denmark
Desmin	1: 200	Ventaan CC1 100 °C	DAKO, Glostrup, Denmark
Myogenin	1: 500	Ventaan CC1 100 °C	DAKO, Glostrup, Denmark
Ki-67	1: 100	Ventana CC1 100 °C	DAKO, Glostrup, Denmark

The tumor cells expressed HMB45, D2–40, GLUT-1, and SMA but were negative for c-kit and CD34, both specific GI stromal tumor markers (GIST) (Fig. 2). Moreover, tumor cells were negative for caldesmon, desmin, myogenin, and Melan-A and S-100. We ruled out leiomyomatosis by HMB45 expression in our tumor and also excluded malignant melanoma by negative for Melan-A and S-100 in our tumor. The Ki-67 labeling index was 10.0%. Immunostaining with D2–40 highlighted endothelial cells lining the slit-like channels. Hence, the lesion was diagnosed as a LAM.

### Preprocessing and analysis of WGS and WTS

Representative tumor areas with at least 90% tumor cell content were outlined for macrodissection on hematoxylin-eosin-stained fresh frozen tissue sections. DNA/RNA extraction was performed from the freshly frozen tissue and patient blood using the Maxwell® RSC DNA/RNA FFPE Kit (Promega, USA).

WGS data were generated using the TruSeq DNA PCR Free library kit and Illumina platform, which had a 150 bp read length, 408 bp fragment length median, and 1,450,195,688 total reads. The paired-end sequence was mapped to the human genome (original GRCh37 from NCBI, February 2009) using Isaac aligner (iSAAC-04.18.11.09). The Isaac aligner identifies and selects the foremost mapping candidates using a 32-mer seed-based search. The 3 end with low quality and adapter sequences were trimmed from the alignment. The Isaac aligner generates a binary alignment output bam file that includes sorted and duplicate-marked data.

WTS data were produced using the SureSelectXT RNA Direct library kit and NovaSeq 6000 platform, creating a 101 bp read length and 161,213,862 total reads. The STAR aligner was used for RNA-Seq data to create a sorted bam file, and its index was created using Samtools v1.9. RSEM [11], with edgeR [12] steps performed to obtain the expression values. The fastq files of the WGS and WTS samples had high Phred quality scores. For tumor WGS, normal blood WGS, and tumor WTS samples, 97.2, 97.6, and 98.42% of the bases had quality scores > 20 for tumor WGS, normal WGS, and tumor WTS samples, respectively.

### Detection of somatic mutation

Somatic mutations for single nucleotide variants (SNVs) and insertions and deletions (INDELs) were detected using the Genome Analysis Toolkit (GATK) Mutect2 v4.1.4.1 [13] with default parameters. All the variants were annotated by ANNOVAR (https://doc-openbio.readthedocs.io/projects/annovar/en/latest/) [14].

**Fig. 3:**
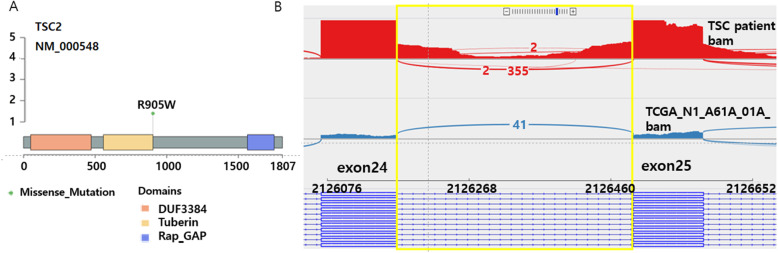
**A** Somatic mutation of in TCS2. One *TSC2* missense somatic mutation (p.R905W) was detected by WGS. **B** Intron region of TCS2 with a novel germline mutation displayed by IGV. The RNA-Seq reads in the intron region with a novel germline mutation next to the acceptor site (chr16:2126489, C > G) are retained between exons 24 and 25. The first and second panels show the intron region of the patient with TSC investigated in this study and a patient with sarcoma from TCGA, respectively. TSC, Tuberous sclerosis complex; WGS, whole-genome sequencing

By employing the WGS data, we detected 12 genes with nonsynonymous somatic mutations and but only 2 mutations (MST1: p.E261Q, and TSC2: p.R905W) are pathogenic (Supplementary Table 1). Among them, *TSC2* had a somatic hotspot mutation (p.R905W, c.C2713T) (Fig. 3A). We assessed whether this deleterious variant in *TSC2* was expressed in mRNA using RNA-Seq data from the tumor sample. STAR 2-PASS [15] was used to create the bam file from the RNA-Seq data, and the reads were visualized using IGV [16]. In total, 570 reads were mapped in the concerned region, and 390 (variant allele frequency [VAF]: 68.0%) had variant sequences.

### Detection of germline mutation

GATK Haplotypecaller [17] was used to identify germline mutations from the WGS bam of normal data. To ensure a high confidence germline mutation, we performed a VariantRecalibrator step of GATK and used the vcf files including dbsnp_138.hg19.vcf [18], hapmap_3.3.hg19.sites.vcf [19], 1000G_omni2.5.hg19.sites.vcf [20], 1000G_phase1.snps.high_confidence.hg19.sites.vcf [21], and Mills_and_1000G_gold_standard.indels.hg19.sites.vcf [22], which were obtained from the GATK resource bundle.

In the present patient with TSC, we detected a novel *TSC2* germline mutation in the *TSC2* intron region, next to the acceptor site (chr16:2126489, C > G). This variant had not been previously reported in ClinVar [23]. We identified that 207 out of 216 reads (VAF: 96%) were mapped to this germline mutation site.

A previously reported mutation at the same intron but into a different nucleotide (NM_000548.4 (*TSC2*):c.2743-3C > A) was observed in assembly GRCh37 (https://www.ncbi.nlm.nih.gov/clinvar/23291215/).

As reads in the RNA-Seq data were mapped to the intron region, iREAD and IRFinder tools were used to detect intron retention. By employing these two tools, intron retention was detected in the chr16:2126258–2,126,491 region, where the germline mutation occurred (Fig. 3B). For comparison, we downloaded the RNA-Seq data of patients with sarcoma from The Cancer Genome Atlas (TCGA) to confirm that this event did not occur randomly. No intron retention was detected in the chr16:2126258–2,126,491 region on analyzing 256 samples with IRFinder (Fig. 3B). We postulate that this intron retention did not appear randomly and that the nearby acceptor site germline mutation might affect the mRNA splicing mechanism.

### Detection of intron retention

iREAD [22] and IRFinder [24] were used to identify intron retention from RNA-Seq fastq data. We used the default parameters for the two tools, except for the entropy score of iREAD. The default threshold was strictly set in the iREAD package. We lowered the entropy score marginally, from 0.9 to 0.85, to detect more candidate IR.

We obtained the trimmed mean of M-values ***(***TMM) of genes with edgeR. The TMM expression value of the *TSC2* gene was 28,576, which was 7.4-fold higher than the average of two standard samples (TMM: 3857).

**Fig. 4:**
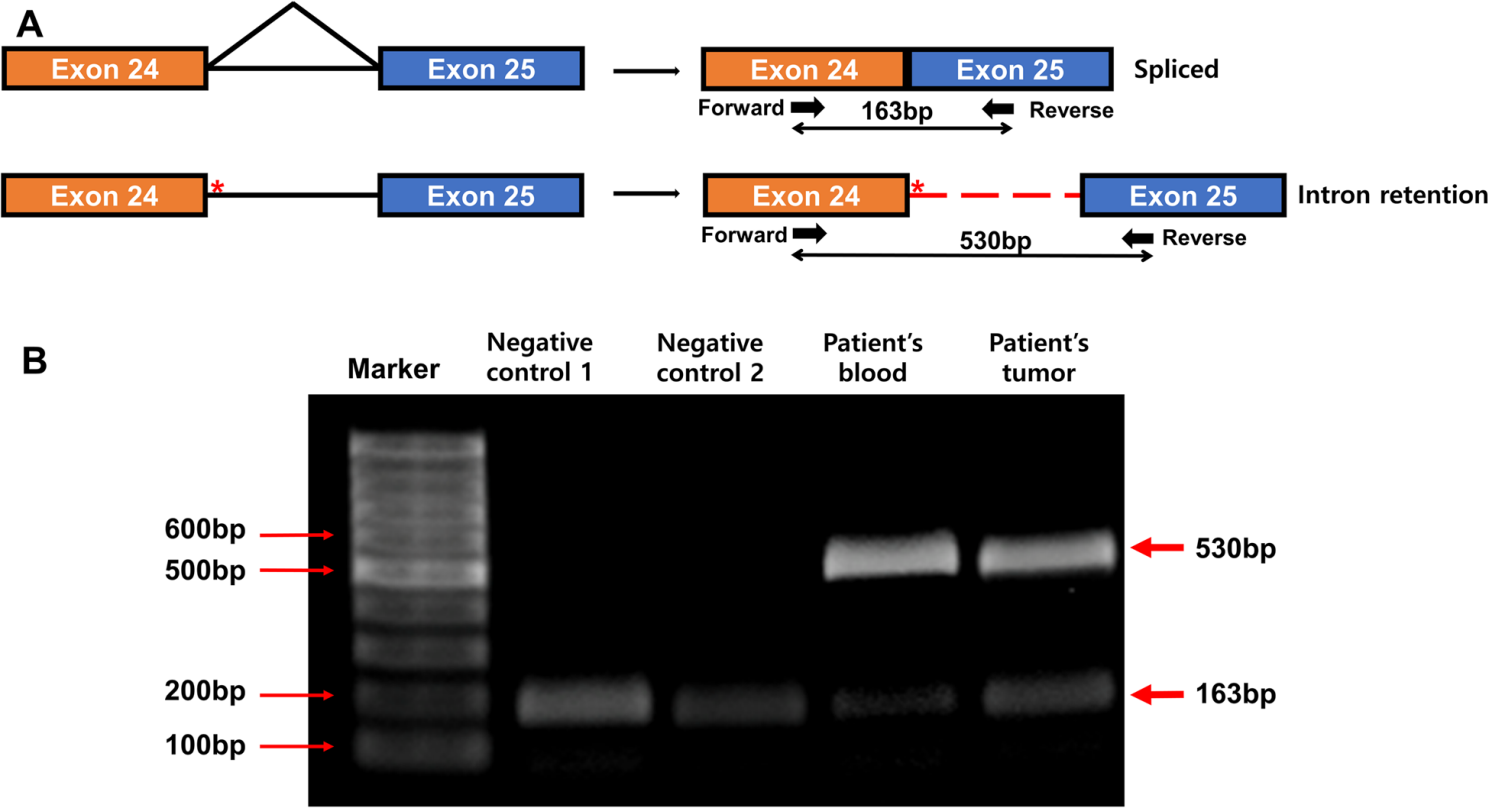
Agarose gel electrophoresis of RT-PCT using primer sets designed to detect TSC2 gene-splicing region of the intron between exon 24 and exon 25 (Forward primer: GTCATAGCCATGTGGTTCA, Reverse primer: TCTTGGGTCTCTCGTTGA). RT-PCR with RNA from our patient’s peripheral blood buffy coat and tumor (lymphangioleiomyoma) revealed the intron retention product at 530 bp and splicing product at 163 pp., but negative controls (RNA from peripheral blood buffy coat of two healthy controls) showed splicing product at 163 bp only

For reverse transcriptase-polymerase chain reaction (RT-PCR), RNA was extracted from the peripheral blood buffy coat and tumor (lymphangioleiomyoma) of this patient and the peripheral blood buffy coat of two healthy (negative) controls. Primer sets were designed to detect TSC2 gene-splicing region in the intron between the exon 24 and exon 25 (Forward primer: GTCATAGCCATGTGGTTCA, Reverse primer: TCTTGGGTCTCTCGTTGA). RT-PCR of this patient’s peripheral blood and tumor RNA revealed the intron retention products at 530 bp and splicing products at 163 bp, but healthy controls showed splicing products at 163 bp only (Fig. 4).

## Discussion and conclusions

TSC is an autosomal dominant disorder caused by genetic alterations in *TSC1* at 9p34 (22%) and *TSC2* at 16p13 (63%), with no mutation identified in about 15% of patients [3]. *TSC1* and *TSC2* encode two tumor suppressor proteins: hamartin and tuberin. Inactivating mutations in either gene fail to inhibit the mTOR pathway and lead to constitutive activation of mTORC1 and dysregulation of downstream molecules, driving uncontrolled cell growth and proliferation; this results in the formation of either hamartomas or benign tumors in multiple organs, including the skin, eyes, heart, kidneys, and brain [3, 7, 25].

LAM is a rare neoplasm that belongs to the family of PEComas [26]. Notably, LAM is six times more common in females than males, and the average age of patients is 42.4 years. The relationship between TSC and LAM is well established. Pulmonary LAM and renal angiomyolipomas are common manifestations of TSC, occurring in 80 and 70% of TSC patients, respectively [1, 27]. Somatic mutations have been detected in sporadic LAMs (https://databases.lovd.nl/shared/diseases/00317).

In patients with TSC, GI manifestations of LAM are infrequent, and only two cases have been reported [28, 29]. One patient had a germline frameshift mutation in *TSC2* (p.K69Nfs*37). The presence of somatic mutations has not been investigated in the remaining patients.

The diagnosis of LAM remains challenging owing to its rarity in extrapulmonary sites [28, 29]. Leiomyomatosis, GIST, and melanoma are the differentials in the present case. Both LAM and leiomyomatosis present as multiple nodules along the bowel walls and show smooth muscle cell proliferation with the expression of smooth muscle markers. GIST comprises spindle and epithelial cells, with c-kit, CD34, or Dog1 expressions. Notably, the melanocytic marker, HMB-45, helps distinguish LAM from leiomyomatosis and GIST, as LAM is 100% immunoreactive for HMB-45, whereas the other two entities are not [30].

In women with TSC, the prevalence of TSC-LAM is 34%; however, the estimated prevalence of S-LAM is approximately 10,000 patients worldwide, which is considerably less than that of TSC-LAM [31, 32]. Both S-LAM and TSC-LAM are associated with mutations in *TSC1* and, more commonly, in *TSC2* genes [33]. Through WGS, we identified a somatic gene mutation, p.R905W (c.C2713T), in the *TSC2* region (of chromosome 16p13). Although this variant has been reported in many individuals with TSC, functional and experimental studies, as well as computational prediction algorithm studies included in ANNOVAR [14], have shown that this mutation is pathogenic as it affects protein function [34]. Arginine at codon 905 is a critical amino acid for the function of tuberin, and two missense mutations, 2714G > A R905Q and 2713C > T R905W in TSC2, have been reported at this codon. Both variations influence the mTOR signaling pathway, affecting cell proliferation, migration, survival, and metabolism [7]. Alteration into different amino acids in the same codon results in varying severity among phenotypes. The R905Q mutation was detected in TSC families with a relatively mild phenotype (normal cognition with no SEGA or epilepsy) [35]. The R905W mutation was found in sporadic TSC and manifested with severe symptoms. According to an article by Sancak et al, all six patients with the R905W mutation presented cortical tubers and SEGA, cognitive impairment, neurologic symptoms, and additional TSC features [36]. Our patient showed a somatic missense mutation (R905W) in LAM and a novel germline intronic mutation (chr16:2126489, C > G), with severe neurological symptoms and multi-organ hamartomas. A known intronic mutation at the same site changed to a different nucleotide (NM_000548.5:c.2743-3C > A, https://www.ncbi.nlm.nih.gov/clinvar/variation/49229/), which affects gene splicing. This variant is not present in population germline databases. When we performed splice site prediction using “Berkeley Drosophila Genome Project, splice site prediction by neural network” [37], this variant was predicted to affect normal splice donor site with the 0.99 prediction score. The novel germline intron mutation found in our case is considered a pathogenic variant with moderate evidence of pathogenicity in TSC.

We provided both RNAseq and RT-PCR confirmation of intron retention. It is justified that the two different methods for the detection of intron retention are consistent. Of note, RNA analysis does not necessarily demonstrate loss of normal TSC2 function, which negatively regulates mTOR signaling. However, intron retention has the potential to cause loss of function, consistent with disease mechanisms.

Recent studies have reported that approximately 20% of PEComas also have a transcription factor E3 (*TFE3*) mutation. PEComas harboring *TFE3* gene rearrangement have been previously reported, presenting an epithelioid appearance, weak or no expression of smooth muscle markers, and robust nuclear staining of TFE3 protein [38, 39]. In the present case, *TFE3* rearrangement was not observed. Moreover, *TFE3* rearrangements and *TSC2* mutations are known to be mutually exclusive [40]. Cutaneous PEComas do not harbor *TFE3* rearrangements, and the occurrence of *TFE3* rearrangement might vary among organs that develop tumors [41].

A novel intron heterozygous mutation in *TSC2* (chr16:2126489, C > G) was detected through WGS and RNA sequencing. Intron retention is an alternative splicing method [42] and plays a regulatory role in neuronal differentiation and neurological diseases [43]. Two different intron retentions of *TSC2* have been reported in patients with TSC (chr16:2106052, C > T and chr16:2126489, C > A) [44, 45]. Different mutations at the same acceptor site of intron have been reported as possibly pathogenic (chr16:2126483,C > G) [46]. Intron retention by intronic mutation of *TSC2* might contribute to our patient’s TSC pathogenesis with multiple hamartomas; however, it remains unclear whether the germline mutation of *TSC2* is pathogenic, given that *TSC2* was overexpressed. As TSC2 is a tumor suppressor, it needs to be underexpressed to be considered a pathogenic mutation. Therefore, further evaluation of the potential biological effects of intron retention in TSC is required.

GI PEComas show a variable spectrum of biological behavior. Folpe et al. have reported that clinically malignant PEComas typically have infiltrative growth patterns, large tumor size (> 5 cm), high nuclear grade, tumor necrosis, increased mitotic activity (> 1/50 HPF), and lymphovascular invasion [30]. A recent study regarding GI PEComas has reported that this tumor demonstrates malignant behavior significantly associated with marked nuclear atypia, diffuse pleomorphism, and mitosis ≥2/10 HPF [47]. The present patient presented a tumor exceeding 5 cm in size, infiltrative growth, tumor necrosis, a mitotic index of 2/50 HPF, and a Ki-67 labeling index of 10%, consistent with malignancy. Additionally, lymph node metastasis was detected. Previous reports have suggested that surgical resection is the best treatment strategy. Considering the genetic etiology of LAM affecting the mTOR pathway, mTOR inhibitors can be a treatment of choice. The mTOR inhibitor rapamycin has several advantages in patients with LAM, stabilizing lung function and improving quality of life. However, the discontinuation of therapy results in disease progression [7, 48].

In conclusion, we presented a case of a TSC patient with extrapulmonary LAM in addition to classic TSC triads. We identified a novel *TSC2* germline intronic mutation (chr16:2126489, C > G) and a second hit somatic *TSC2*-mutation in exon 24 (p.R905W, c.C2713T) in the patient’s blood and tumor samples. LAM diagnosis and treatment can be challenging; nevertheless, it is essential to be aware of the association between *TSC2* mutations and LAM. Our findings provide clues for the repair and prevention of TSC and suggest that intron retention by intronic mutation of *TSC2* is sufficient to develop severe TSC manifestations; however, the development of LAM requires an additional somatic oncogenic mutation. Future multi-omics studies need to elucidate the precise pathogenesis and tumorigenesis of TSC and establish a suitable treatment strategy for this disease.

## Supplementary Information

**Additional file 1: Supplementary Table 1**. Somatic nonsynonymous single nucleotide variation detected by WGS in our lymphangioleiomyoma, but most were VUS except MST1 and TSC2.

## Data Availability

The data that support the findings of this study are available from the corresponding authors (Hyunju Lee and Sung-Hye Park) upon reasonable request.

## Abbreviations

CT: Computed tomography
GIST: Gastrointestinal stromal tumor markers
LAM: Lymphangioleiomyomatosis
MITF: Melanocyte inducing transcription factor/microphthalmia transcription factor
MRI: Magnetic resonance imaging
PEComa: Perivascular epithelioid cell tumor
SEGA: Subependymal giant cell astrocytoma
TSC: Tuberous sclerosis complex
WGS: Whole-genome sequencing
WTS: Whole transcriptome sequencing
