# Supplementary material for: A second hit somatic (p.R905W) and a novel germline intron-mutation of TSC2 gene is found in intestinal lymphangioleiomyomatosis: a case report with literature review
Source: Diagn Pathol. 2021 Aug 31;16:83. doi: 10.1186/s13000-021-01138-8 (PMC8406734; doi:10.1186/s13000-021-01138-8)
Supplement: Supplementary file 1 — Additional file 1: Supplementary Table 1. Somatic nonsynonymous single nucleotide variation detected by WGS in our lymphangioleiomyoma, but most were VUS except MST1 and TSC2. [file 13000_2021_1138_MOESM1_ESM.docx]

Supplementary Table 1.  Somatic nonsynonymous single nucleotide variation detected by WGS in our lymphangioleiomyoma, but most were VUS except MST1 and TSC2.

|  | **Gene** | **Chr** | **Position** | **Ref** | **Var** | **Coding change** | **Allele frequency** | **Cosmic  (FATHMM prediction score)** |
| --- | --- | --- | --- | --- | --- | --- | --- | --- |
| 1 | NBPF19 | chr1 | 144190689 | A | T | p.Q1021L | 7/216 (3.2%) | Absent (VUS) |
| 2 | NBPF26 | chr1 | 148006375 | A | G | p.V1170A | 10/140 (7.1%) | Absent (VUS) |
| 3 | ANKRD36 | chr2 | 97911486 | A | G | p.Q1695R | 68/125 (54.4%) | Absent (VUS) |
| 4 | MST1 | chr3 | 49724183 | C | G | p.E261Q | 11/70 (15.7%) | Somatic, pathogenic (score: 0.78) |
| 5 | NECTIN3 | chr3 | 110831093 | A | T | p.Y126F | 5/74 (6.7%) | Somatic, but neutral (score: 22) |
| 6 | USP17L20 | chr4 | 9269838 | T | C | p.L165P | 9/252 (3.5%) | Absent (VUS) |
| 7 | POM121 | chr7 | 72412591 | G | A | p.A422T | 9/53 (16.9%) | Somatic, but neutral (score: 0.01) |
| 8 | ANKRD20A4 | chr9 | 69422031 | T | C | p.W499R | 10/61 (16.3%) | Somatic, but neutral (score: 0.00) |
| 9 | GOLGA6L1 | chr15 | 22743461 | T | G | p.W616G | 9/92 (9.7%) | Somatic, but neutral (score: 0.00) |
| 10 | TSC2 | chr16 | 2126142 | C | T | p.R905W | 35/81 (43.2%) | Somatic, pathogenic (score: 0.93) |
| 11 | FTCD | chr21 | 47565413 | C | T | p.R393H | 32/87 (36.7%) | Somatic, n/a (VUS) |
| 12 | TEX13D | chrX | 123468656 | G | A | p.G630D | 6/90 (6.6%) | absent (VUS) |

VUS: variant of unknown significance, n/a: not available.
